# Treatment of extracranial rhabdoid tumor with intensive ifosfamide-containing chemotherapy regimens

**DOI:** 10.3389/fonc.2026.1785130

**Published:** 2026-04-15

**Authors:** Jillian P. Smith, Liny John, Aerang Kim, Holly J. Meany, Amy B. Hont, Amy Frantz, Jeffrey S. Dome

**Affiliations:** Department of Pediatrics, George Washington University School of Medicine and Health Sciences, Division of Pediatric Oncology, Children’s National Hospital, Washington, DC, United States

**Keywords:** cyclophosphamide, ifosfamide, malignant rhabdoid tumor (MRT), rhabdoid tumor, rhabdoid tumor of the kidney (RTK)

## Abstract

**Introduction:**

Extracranial malignant rhabdoid tumors (MRT) and rhabdoid tumors of the kidney (RTK) are rare pediatric solid tumors characterized by aggressive behavior and poor prognosis. Case reports have demonstrated successful outcomes using ifosfamide-containing chemotherapy regimens, leading some clinicians to incorporate this alkylating agent into treatment plans. We assessed our institutional experience treating MRT and RTK using ifosfamide-containing regimens.

**Methods:**

Fifteen patients with histologically confirmed MRT or RTK and loss of INI1 who were treated at Children’s National Hospital (CNH) between 2009 and 2025 were reviewed retrospectively.

**Results:**

The median age at diagnosis was 16 months (range: 3–171 months). Eleven patients (73.3%) had MRT and four (26.7%) had RTK. Treatment involved multimodal therapy, including surgical resection when feasible, radiation therapy, and intensive chemotherapy. Eleven patients received regimens including ifosfamide, in some cases with higher-dose cyclophosphamide compared to recent Children’s Oncology Group and European Soft Tissue Sarcoma Study Group protocols. Three-year event-free survival (EFS) and overall survival (OS) were 42.4% (95% CI: 22.7-79.3%) and 40.4% (95% CI: 20.8-78.5%), respectively. No significant difference in 3-year EFS was observed with ifosfamide (43.6%, 95% CI: 21.8-87.4%) versus without ifosfamide (37.5%, 95% CI: 8.4-100%; p=0.85). Second and third-line treatment regimens included tazemetostat (n=3), temozolomide/irinotecan +/- vincristine (n=3), alisertib (n=1), and cemiplimab (n=1); however, no responses were observed.

**Conclusion:**

Ifosfamide-containing regimens did not appear to improve outcomes for MRT or RTK. Novel, targeted therapeutic strategies are urgently needed to improve survival for patients with rhabdoid tumor, particularly those with stage III/IV disease.

## Introduction

Rhabdoid tumors (RT) are rare, highly aggressive pediatric solid tumors that typically present in early childhood. These tumors most commonly arise in the kidneys (RTK), soft tissues (malignant RT [MRT]), or the central nervous system, where they are classified as atypical teratoid/rhabdoid tumors (AT/RT) (1–5). Most RT are associated with pathogenic variants in *SMARCB1* (also known as INI1 and SNF5) a tumor suppressor gene on chromosome 22q. INI1 protein is a component of the SWI/SNF chromatin remodeling complex and plays a critical role in tumor suppression (4, 5). Germline mutations in *SMARCB1* have been implicated in hereditary rhabdoid tumor predisposition syndrome (6), and loss of INI1 expression is a defining histologic feature of MRT and RTK (7).

Despite advances in the treatment of pediatric renal and extrarenal solid tumors, the prognosis for individuals with MRT and RTK remains poor, with 4-year overall survival (OS) rates ranging from 23.3-33% (8–12). Outcomes are particularly unfavorable for patients with advanced-stage disease at presentation or for those diagnosed before six months of age (13). Treatment is typically multimodal, including surgical resection, chemotherapy, and/or radiation therapy, although no standardized regimen exists given the relative rarity of these tumors. Recent reports of clinical trials for patients with extra-cranial rhabdoid tumor from the Children’s Oncology Group (COG) and the European Soft Tissue Sarcoma Study Group (EPSSG) demonstrated an event-free survival (EFS) of 23-32% and OS of 32-38% using regimens of cyclophosphamide/carboplatin/etoposide (CyCE) alternating with vincristine/doxorubicin/cyclophosphamide (VDCy) (8, 10). A report from the German Society of Pediatric Hematology and Oncology (GPOH) indicated that high-dose therapy and stem cell rescue does not seem to improve outcomes for RTK (14). Currently, there is no standard-of-care for relapsed or refractory MRT or RTK.

Case reports of treatment successes for patients with RT using ifosfamide-containing regimens (15–17) and case series demonstrating improved outcomes with relatively high doses of cyclophosphamide (18) have led physicians at Children’s National Hospital (CNH) to make adjustments to the COG and EPSSG approaches for rhabdoid tumor. To better characterize the outcomes of patients treated for RTK or MRT with such dose-intensive chemotherapy, we conducted a retrospective review of cases treated at CNH between 2009 and 2025.

## Materials and methods

### Patients

This retrospective chart review was approved by the CNH Institutional Review Board (STUDY00001747). Fifteen patients with MRT or RTK treated at CNH between January 1, 2009 and December 1, 2025 were identified and reviewed. All patients had a confirmed diagnosis of RT based on histologic criteria, and all patients had documented loss of INI1 by immunohistochemistry or cytogenetics.

Data collected included demographic characteristics, primary tumor site, stage of disease, presence of metastases, tumor molecular features, first-line treatment details, regimens for relapsed or refractory disease, and clinical outcomes. For disease staging at diagnosis, the COG Renal Tumor Staging System was used for RTK (19), and the Intergroup Rhabdomyosarcoma Study Group (IRSG) Grouping System was used for MRT (20). Treatment modalities included chemotherapy, surgery, radiation, and immunotherapy. Initial staging evaluation and treatment responses were evaluated using magnetic resonance imaging (MRI), computed tomography (CT), or fluorodeoxyglucose (FDG) positron emission tomography (PET). Responses were defined using Response Evaluation Criteria In Solid Tumors (RECIST) guidelines (21). Complete response (CR) was defined as absence of active disease on imaging, partial response (PR) as ≥ 30% reduction in tumor size as measured by sum of longest diameter, progressive disease (PD) as an increase in primary tumor size of ≥ 20% from nadir or the appearance of new lesions, and stable disease (SD) was defined as neither PR nor PD from the primary tumor site nadir.

### Treatment regimens

Regimens included VDCy/CyCE (V: vincristine, D: doxorubicin, Cy: cyclophosphamide, C: carboplatin, E: etoposide) using doses from the EpSSG-2005 and AREN0321 studies (8, 10); VDCy/ICE (I: ifosfamide); or ifosfamide and doxorubicin plus CyCE. Ifosfamide dosing ranged from 1800–3000 mg/m^2^, and high dose cyclophosphamide was defined as dosing greater than or equal to 2100 mg/m^2^/dose.

### Statistical analysis

OS was calculated from the date of pathology-confirmed diagnosis to either date of death or last documented follow up as of December 1, 2025. EFS was calculated from the date of pathology-confirmed diagnosis to date of relapse, disease progression, second malignancy, death, or last documented follow up if no events occurred. The Kaplan-Meier method was utilized to generate survival estimates for EFS and OS using GraphPad Prism version 10 (Dotmatics) and R software (R Core Team, 2025). Survival curves were analyzed using Mantel-Cox log-rank tests. Significance level was set at p<0.05.

## Results

### Eligibility and patient characteristics

Fifteen patients had RT confirmed by histology demonstrating classic features of small round blue cells with hyperchromatic nuclei, coarse chromatin, and loss of INI1 expression. Demographic characteristics are described in **Table 1**. The median age at diagnosis was 16 months (range: 3–171 months). Nine patients (60%) were diagnosed before two years of age and six (40%) were diagnosed at or after two years of age.

**Table 1:** Demographic and diagnostic characteristics of entire cohort (n=15).

Sex	
Male	8 (53.3%)
Female	7 (46.7%)
Race	
White	5 (33.3%)
Black	2 (13.3%)
Asian	1 (6.7%)
Multiple	1 (6.7%)
Unknown	6 (40%)
Ethnicity	
Non-Hispanic	4 (26.7%)
Hispanic	3 (20%)
Unknown	8 (53.3%)
Age at diagnosis	
< 2 years	9 (60%)
≥ 2 years	6 (40%)
Primary tumor location	
Extrarenal	11 (73.3%)
Liver	3 (20%)
Mediastinum	2 (13.3%)
Superficial soft tissue	2 (13.3%)
Eye	1 (6.7%)
Ovary	1 (6.7%)
Paravertebral	1 (6.7%)
Pericardium and mediastinum	1 (6.7%)
Renal	4 (26.7%)
Left kidney	2 (13.3%)
Right kidney	2 (13.3%)
Bilateral kidneys	0
Stage (RTK) or group (MRT) at diagnosis	
I	2 (13.3%)
II	3 (20%)
III	4 (26.7%)
IV	6 (40%)
Metastases	
Yes	6 (40%)
Lungs	3 (20%)
Lungs, mediastinal LN	1 (6.7%)
Anterior chest wall	1 (6.7%)
Liver, lungs, retroperitoneum	1 (6.7%)
No	9 (60%)

LN, lymph node; RTK, rhabdoid tumor of the kidney; MRT, malignant rhabdoid tumor. Staging for RTK per COG Renal Tumor Staging System, group for MRT per IRSG Grouping System.

Primary tumor sites included extrarenal locations in 11 patients (73.3%) including the liver (n=3, 20%), mediastinum (n=2, 13.3%), superficial soft tissue (hand and axilla, back; n=2, 13.3%), eye and orbit (n=1, 6.7%), ovary (n=1, 6.7%), paravertebral region (n=1, 6.7%), and pericardium and mediastinum (n=1, 6.7%). Four patients (26.7%) had RTK, all of whom had unilateral disease. Metastatic disease at diagnosis was present in six patients (40%). All patients had confirmed loss of INI1 by histology. Stage or group at diagnosis was stage/group I for two patients (13.3%), stage/group II for three patients (20%), stage/group III for four patients (26.7%), and stage/group IV for six patients (40%). Five patients (P5, P6, P11, P12, P15) underwent testing for *SMARCB1* germline mutations, of which one patient (P12) was heterozygous for a pathogenic frameshift mutation in *SMARCB1*.

### Treatment

**Figure 1:**
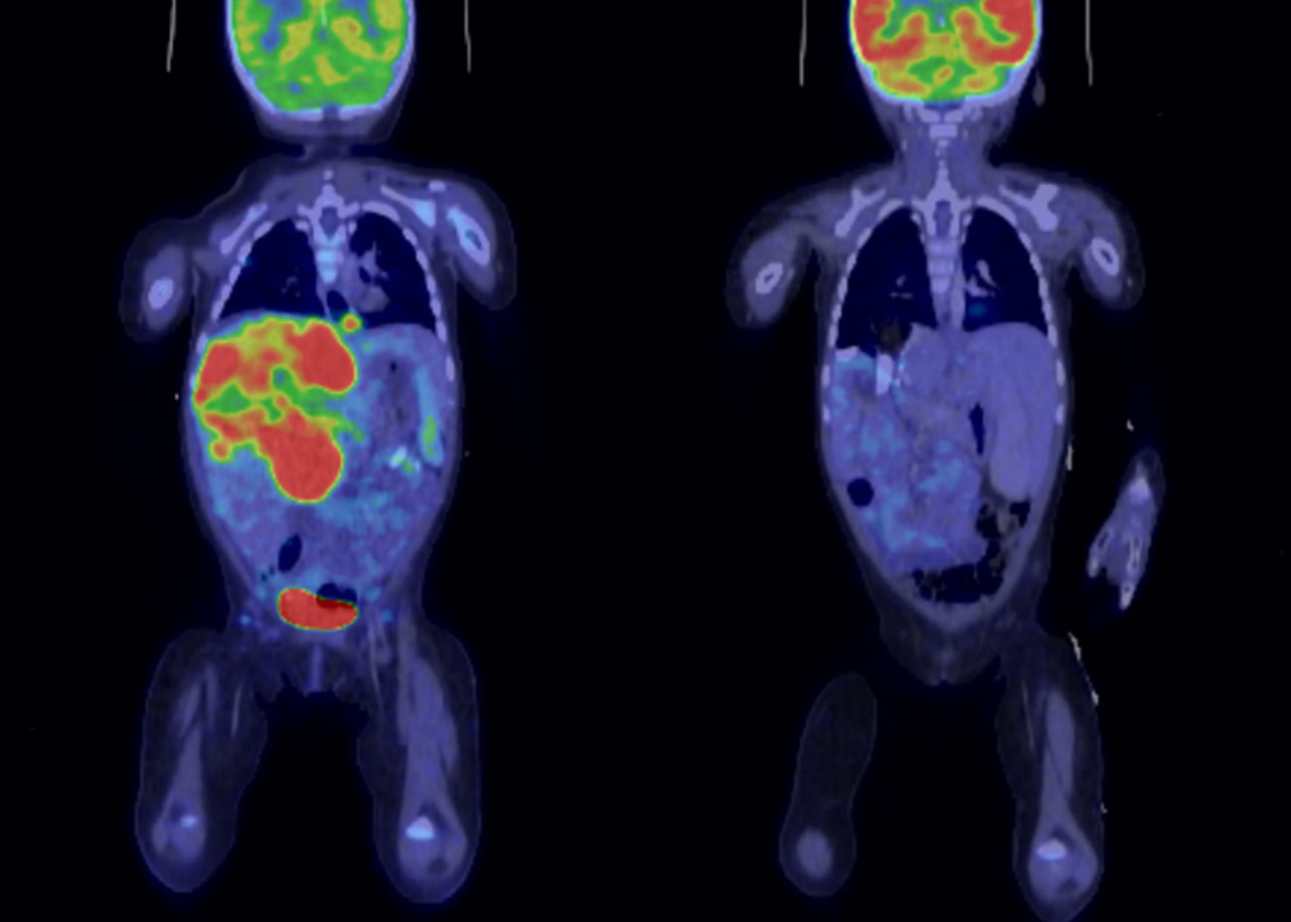
For patient P11 with extra-renal rhabdoid tumor (liver), fluorodeoxyglucose positron emission tomography (FDG-PET) **(A)** at diagnosis with FDG avid hepatic lesion; **(B)** status post hepatic lobe resection and eight cycles of VDCy/ICE showing response with small amount of residual FDG uptake at the surgical margins and no distinct mass identified.

Treatment details are summarized in **Table 2**. All 15 patients received chemotherapy as part of the initial treatment. Regimens included VDCy/ICE (vincristine, doxorubicin, cyclophosphamide, ifosfamide, carboplatin, etoposide; n=10), VDCy/CyCE (vincristine, doxorubicin, cyclophosphamide, carboplatin, etoposide; n=4), or ifosfamide and doxorubicin combined with CyCE (cyclophosphamide, carboplatin, etoposide; n=1). Seven patients underwent an upfront complete resection of their primary mass (46.7%), seven had an upfront biopsy only (46.7%), and one patient underwent a partial excision upfront (6.7%). Radiation therapy was administered to 10 patients (66.7%) using either conventional radiation therapy or proton-beam radiation therapy, with a median time from diagnosis to start of radiation of 4.5 months (range: 20 days-9 months). Five patients (33.3%) did not receive radiation. Eight patients (53.3%) underwent their first therapeutic intervention of surgical resection at diagnosis. Six patients (40%) started treatment with chemotherapy a median of 6 days after diagnosis (range: 2–11 days), and one patient was diagnosed at an outside facility and thus duration of time to start of chemotherapy is unknown. Of the seven patients who did not have upfront surgical resection, four received radiation therapy at a median time from diagnosis to start of radiation of 6.25 months (range: 4–9 months). Best treatment response was assessed by comparing baseline imaging (MRI, CT, or FDG-PET) with post-treatment studies as shown for patient P11 (**Figure 1**), who demonstrated partial response following hepatic lobe resection and chemotherapy with residual uptake in the tumor bed.

**Table 2:** Clinical characteristics, treatment regimens, and outcomes.

Patient number	Sex	Age at diagnosis (months)	Primary site	Metastases	Stage (RTK) or group (MRT)	Loss of INI1	Additional cytogenetic features	Chemotherapy (dose per cycle in mg/kg/dose or mg/m^2^/dose)	Upfront surgery	Radiation location (dose in cGy)	Second surgery	Best response by RECIST criteria	Vital status (months after diagnosis)
P1	F	172	Paravertebral	Lungs	IV	Loss		Ifosfamide, Doxorubicin x 5 cycles; CyCE x 3 cycles (I 3000 mg/m^2^/dose days 1-3; D 37.5 mg/m^2^/dose days 1-2; Cy 440 mg/m^2^/dose days 1-4; C*day 1; E 100 mg/m^2^/dose days 1-4)	Partial	Primary site	—	PR	Unknown (10)
P2	M	13	Kidney, L	Liver, lungs, retroperitoneum	IV	Loss		VDCy x 3 cycles; CyCE x 2 cycles (V 0.05 mg/kg/dose days 1, 8, 15; D 45 mg/m^2^/dose day 1; Cy 440 mg/m^2^/dose days 1-4; Cy 1200 mg/m^2^/dose day 1; C* day 1; E 100 mg/m^2^/dose days 1-4)	Biopsy	—	—	PR	DOD (3)
P3	F	81	Ovary, L	None	III	Loss	Homozygous SMARCB1 deletion	VDCy x 5 cycles; ICE x 5 cycles (V 1.5 mg/m^2^/dose days 1, 8, 15; D 37.5 mg/m^2^/dose days 1-2; Cy 1200 mg/m^2^/dose days 1-2; I 1800 mg/m^2^/dose days 2-5; C 635 mg/m^2^/dose day 1; E 100 mg/m^2^/dose days 2-5)	Complete	Whole abdomen (4860 cGy), primary site (3600 cGy)	—	CR	NED (40)
P4	M	3	Kidney, R	None	III	Loss		VDCy x 5 cycles; ICE x 3 cycles (V 0.025 mg/kg/dose days 1, 8, 15; D 12.5 mg/kg/dose days 1-2; Cy 40 mg/kg/dose days 1-2; I 66.7 mg/kg/dose days 2-4; C* day 1; E 3.33 mg/kg/dose days 2-4)	Complete	Whole abdomen, R flank (1950 cGy)	—	CR	DOD (21)
P5	M	63	Pericardium, mediastinum	None	II	Loss		VDCy x 4 cycles; ICE x 4 cycles (V 1.5 mg/m^2^/dose days 1, 8, 15; D 45 mg/m^2^/dose day 1; Cy 2100 mg/m^2^/dose days 1-2; I 2000 mg/m^2^/dose days 1-3; C* day 1; E 100 mg/m^2^/dose days 1-3)	Complete	Mediastinum (4500 cGy)	—	CR	NED (87)
P6	F	16	Kidney, R	None	III	Loss		VDCy x 4 cycles; ICE x 4 cycles (V 0.05 mg/kg/dose days 1, 8, 15; D 1.25 mg/kg/dose days 1-2; Cy 60 mg/kg/dose day 1; I 66.7 mg/kg/dose days 1-3; C* day 1; E 3.33 mg/kg/dose days 1-3)	Complete	Whole abdomen	—	CR	Secondary malignancy (116)
P7	M	70	Orbit, Eye	None	II	Loss		VDCy x 4 cycles; CyCE x 5 cycles (V 1.5 mg/m^2^/dose days 1, 8, 15; D 45 mg/m^2^/dose day 1; Cy 1200 mg/m^2^/dose day 1; Cy 440 mg/m2/dose days 1-4; C* day 1; E 100 mg/m^2^/dose days 1-4)	Complete	Primary site (4500 cGy)	—	CR	NED (45)
P8	F	47	Mediastinum	None	III	Loss		VDCy x 5 cycles; CyCE x 5 cycles (V 1.5 mg/m^2^/dose days 1, 8, 15; D 45 mg/m^2^/dose day 1; Cy 1200 mg/m^2^/dose day 1; Cy 440 mg/m2/dose days 1-4; C* day 1; E 100 mg/m^2^/dose days 1-4)	Biopsy	Primary site (4500 cGy)	Complete	CR	DOD (12)
P9	M	78	Mediastinum	None	I	Loss		VDCy x 5 cycles; CyCE x 3 cycles (V 1.5 mg/m^2^/dose days 1, 8, 15; D 45 mg/m^2^/dose day 1; Cy 1200 mg/m^2^/dose day 1; Cy 440 mg/m^2^/dose days 1-4; C* day 1; E 100 mg/m^2^/dose days 1-4)	Biopsy	Primary site (4500 cGy)	Complete	CR	Unknown (5)
P10	M	12	Liver	Chest wall	IV	Loss		VDCy/ICE	Biopsy	Whole abdomen	Partial (liver mass resection)	CR	DOD (14)
P11	F	13	Liver	Lungs, mediastinal LN	IV	Loss	RET mutation T636M, SMARCB1 loss	VDCy x 4 cycles; ICE x 4 cycles (V 0.05 mg/kg/dose day 1; D 45 mg/m^2^/dose day 1; Cy 2100 mg/m^2^/dose days 1-2; I 2000 mg/m^2^/dose days 1-3; C* day 1; E 100 mg/m^2^/dose days 1-3)	Biopsy	Primary site (3600 cGy)	Complete	PR	DOD (13)
P12	F	19	Kidney, L	None	II	Loss		VDCy x 4 cycles; ICE x 4 cycles (V 0.05 mg/kg/dose days 1, 8, 15; D 37.5 mg/m^2^/dose days 1-2; Cy 2100 mg/m^2^/dose days 1-2; I 2000 mg/m^2^/dose days 1-3; C* day 1; E 100 mg/m^2^/dose days 1-3)	Complete	—	—	CR	DOC (7)
P13	M	9	Liver	Lungs	IV	Loss		VDCy x 2 cycles; ICE x 1 cycles (V 0.025 mg/kg/dose days 1, 8, 15; D 1.25 mg/kg/dose days 1-2; Cy 40 mg/kg/dose day 1; I 65 mg/kg/dose days 1-3; C† day 1; E 3.3 mg/kg/dose days 1-3)	Biopsy	—	—	SD	DOD (2)
P14	M	4	Hand, axilla	Lungs	IV	Loss		VDCy x 1 cycle; ICE x 1 cycle (V 0.025 mg/kg/dose days 1, 8, 15; D 1.5 mg/kg/dose day 1; Cy 40 mg/kg/dose day 1; I 65 mg/kg/dose days 1-3; C† day 1; E 3.3 mg/kg/dose days 1-3)	Biopsy	—	—	PD	DOD (3)
P15	F	4	Back	None	I	Loss	22q del	VDCy x 4 cycles; ICE x 4 cycles (V 0.025 mg/kg/dose days 1, 8, 15; D 1.2 mg/kg/dose days 1-2; Cy 40 mg/kg/dose day 1; I 65 mg/kg/dose days 1-3; C† day 1; E 3.3 mg/kg days 1-3)	Complete	—	—	CR	NED (198)

F, female; M, male; L, left; R, right; del, deletion; V, vincristine; D, doxorubicin; Cy, cyclophosphamide; I, ifosfamide; C, carboplatin; E, etoposide; CyCE, Cyclophosphamide, carboplatin, etoposide; VDCy, vincristine, doxorubicin, cyclophosphamide; ICE, ifosfamide, carboplatin, etoposide; PR, partial response; CR, complete response; PD, progressive disease; DOD, died of disease; NED, no evidence of disease; DOC, died of complication. Carboplatin dosed per glomerular filtration rate (GFR, mL/min/1.73 m^2^) as measured by technetium 99-diethylenetriaminepentaacetic acid (Tc-DTPA) serum clearance. C*: carboplatin dosing by nomogram as follows: GFR > 150 mL/min/1.73 m^2^: C 560 mg/m^2^ (18.7 mg/kg if < 1 year of age or < 10 kg); GFR 100–150 mL/min/1.73 m^2^: C 500 mg/m^2^ (16.6 mg/kg); GFR 75–99 mL/min/1.73 m^2^: C 370 mg/m^2^ (12.3 mg/kg); GFR 50–74 mL/min/1.73 m^2^: C 290 mg/m^2^ (9.7 mg/kg); GFR 30–49 mL/min/1.73 m^2^: C 200 mg/m^2^ (6.7 mg/kg); GFR < 30 mL/min/1.73 m^2^: hold carboplatin. C^†^, carboplatin dosing by Calvert formula targeting area under the curve (AUC) of 6 mg/mL per minute, dose (mg/m^2^)=6(AUC)x[[0.93 xGFR (mL/min/m^2^)]+15].

### Response to therapy

The best response to therapy was CR in 10 patients (66.7%), PR in three patients (20%), SD in one patient (6.7%), and PD in one patient (6.7%). CR was achieved after surgery in four patients (4/10, 40%); chemotherapy and surgery in one patient (1/10, 10%); and chemotherapy, surgery and radiation therapy in five patients (5/10, 50%).

### Outcomes

The median follow-up was 13 months (range: 2–198 months) (**Table 1**). Eight patients (53.3%) died during or after first-line therapy; seven (46.7%) from disease progression or recurrence and one (6.7%) from infectious complications. All four patients with PD had progression at the site of the primary mass, and growth of existing metastases and/or new metastases. Of the three patients with recurrent disease, all had recurrence at the primary site as well as new metastases. Four patients (26.7%) had no evidence of disease at last follow-up, and one patient (6.7%) developed a secondary malignancy (chondrosarcoma) 9.5 years after completion of therapy, suspected to be related to radiation therapy. Two patients (13.3%) were lost to follow up 10 months (P1) and five months (P9) after diagnosis, and outcomes for these patients are reported as of the last documented visit.

**Figure 2:**
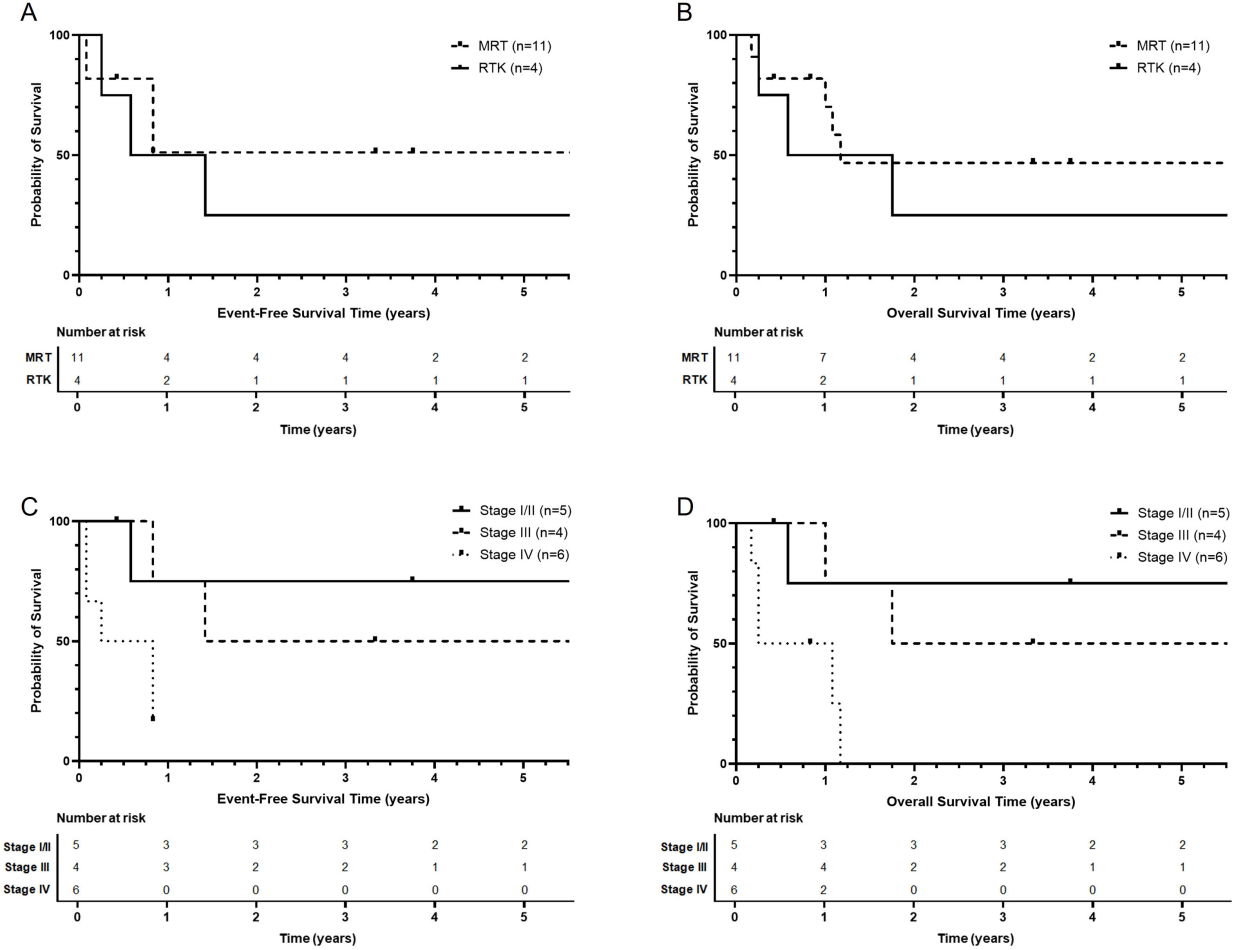
Event-free survival **(A)** and overall survival **(B)** of patients with extracranial MRT compared to patients with RTK as measured in years since diagnosis. Event-free survival **(C)** and overall survival **(D)** of patients by stage as measured in years since diagnosis.

Three-year EFS was 42.4% with a median EFS of 17 months (95% CI: 22.7-79.3%) (**Figure 2**). Three-year OS was 40.4% with a median OS of 14 months (95% CI: 20.8-78.5%). Among patients with MRT (n=11), 3-year EFS was 51.1% (95% CI: 27.9-93.6%) and 3-year OS was 46.8% (95% CI: 23.2-94.1%). For RTK, 3-year EFS was 25% (95% CI: 4.6-100%) and 3-year OS was 25% (95% CI: 4.58-100%). Three-year EFS was 75% (95% CI: 42.6-100%) for patients with stage or group (stage/group) I or II disease (I/II), 50% for stage/group III (95% CI: 18.8-100%), and 0% for stage/group IV. Groupwise comparisons of 3-year EFS were as follows: stage/group I/II versus III (p=0.352), stage/group III versus IV (p=0.062), and stage I/II versus IV (p=0.078). Three-year OS for patients with stage/group I/II disease at diagnosis was 75% (95% CI: 42.6-100%), 50% for stage/group III (95% CI: 18.8-100%), and 0% for stage/group IV. Groupwise comparisons of 3-year OS were as follows: stage/group I/II versus III (p=0.62), stage/group III versus IV (p=0.045), and stage/group I/II versus IV (p=0.042).

Three-year EFS for patients who were diagnosed at age < 2 years was 22.2% (95% CI: 6.6-75.4%), compared to 80% (95% CI 51.6-100%) for those ≥ 2 years (p=0.042). Three-year OS for patients who were diagnosed at age < 2 years was 22.2% (95% CI: 6.6-75.4%), compared to 75% (95% CI: 42.6-100%) for those age ≥ 2 years (p=0.076).

When comparing patients who received chemotherapy regimens including ifosfamide (n=11) to patients receiving regimens without ifosfamide (n=4), no significant difference in 3-year EFS with ifosfamide (43.6%, 95% CI: 21.8-87.4%) versus without ifosfamide (37.5%, 95% CI: 8.4-100%; p=0.85) or OS with ifosfamide (41.6%, 95% CI 19.9-86.8%) versus without ifosfamide (37.5%, 95% CI 8.4-100%; p=0.774) was observed. Of the 11 patients who received either ifosfamide-containing regimens +/- higher-dose cyclophosphamide compared to the AREN0321 and EpSSG-2005 studies (P1, P3, P4, P5, P6, P10, P11, P12, P13, P14, P15), one was lost to follow-up (initial group IV), three were alive without evidence of disease (initial stage/group I, II, III), one had a second malignancy nine years after completion of therapy for rhabdoid tumor (initial stage/group III), five died of disease (initial stage/group III [n=1] and IV [n=4]), and one died of a complication of therapy (initial stage/group II). Of the four patients who did not receive ifosfamide or higher-dose cyclophosphamide, one was lost to follow-up (initial stage/group I), one was alive without evidence of disease (initial stage/group II), and two died of disease (initial stage/group III, IV).

### Second- and third-line treatment

Progressive or relapsed disease occurred in patients P2, P4, P8, P10, P11, P13, and P14 (n=7/15, 46.7%), and one patient (P6) developed a secondary malignancy (6.7%). The median time from diagnosis to progression or relapse was 10 months (range: 1–17 months). Five patients went on to receive second-line treatment including the EZH2 inhibitor tazemetostat (n=2); vincristine, irinotecan, and temozolomide (VIT, n=2); or irinotecan and temozolomide (n=1) (**Table 3**). P14 also underwent a palliative left upper extremity amputation for disease control. Of the five patients who received second-line chemotherapy or biological therapy, two died of disease during treatment. Three went on to third-line regimens including the PD-1 inhibitor cemiplimab, tazemetostat, and the Aurora-A kinase inhibitor alisertib. No objective responses to second or third-line chemotherapy were seen, and all five patients who received such therapy died from disease progression.

**Table 3:** Relapsed/refractory regimens.

Patient number	Months from diagnosis to relapse or progression	Location of relapse or progression	Second-line therapy					Third-line therapy				
			Chemotherapy or immunotherapy	Surgery	Duration of treatment (months)	Status at end of therapy	Best response by RECIST criteria	Chemotherapy or immunotherapy	Surgery	Duration of treatment (months)	Status at end of therapy	Best response by RECIST criteria
P4	17	R renal fossa, liver	Tazemetostat	—	2	PD	PD	Cemiplimab	—	2	DOD	—
P8	10	Spine, leptomeninges	Temozolomide, irinotecan	—	1	DOD	—	—	—	—	—	—
P10	10	Progression at primary site (liver), abdomen	Tazemetostat	—	2	PD	PD	Alisertib	—	2	DOD	—
P11	10	Lungs, mediastinum	VIT	—	1	PD	PD	Tazemetostat	—	2	DOD	PD
P14	1	Progression at primary site (L hand)	VIT	Palliative LUE amputation	—	DOD	PD	—	—	—	—	—

L, left; R, right; VIT, vincristine, irinotecan, temozolomide; LUE, left upper extremity; PD, progressive disease, DOD, died of disease.

## Discussion

Treatment of MRT and RTK remains a challenge despite intensive multimodal treatment regimens. The present study indicates that ifosfamide-containing regimens with or without higher-dose cyclophosphamide did not demonstrate measurable improvement compared to regimens used in the COG AREN0321 and EPSSG-Soft Tissue Sarcoma-2005 studies. The EPSSG study evaluated VDCy (cyclophosphamide 1200 mg/m^2^/dose x 1 day)/Cy (cyclophosphamide 440 mg/m^2^/dose x 5 days)-CE and demonstrated three-year OS of 38.4% (8). The COG AREN0321 study used the same regimen (UH-1) as EPSSG-2005, resulting in a similar 4-year OS of 30.6% (10). Case reports of ifosfamide-containing regimens have demonstrated successful outcomes for high-stage rhabdoid tumors (15–17). Additionally, VDCy/ICE was shown to be safe and tolerable in a retrospective review of high-risk renal tumors, with three of nine patients with RT alive without disease and three additional patients alive with disease (22). In our series, outcomes with ifosfamide-containing regimens were no better than regimens containing only cyclophosphamide. Although the small sample size limits drawing definitive conclusions about the relative value of ifosfamide, the data indicate that ifosfamide does not provide a substantial benefit.

Likewise, a report showed that patients with MRT treated since 2002 who generally received higher-dose cyclophosphamide (2100 mg/m^2^/day x 2 days) with vincristine and doxorubicin had superior outcomes compared to patients treated with a lower-dose alkylator before 2002 (18). In our series, of three patients who received this cyclophosphamide dose, one was alive with no evidence of disease, one died of disease, and one died of complications.

Five patients in this series received targeted therapy as second- or third-line therapy: three with the EZH2 inhibitor tazemetostat, one with the immune checkpoint inhibitor cemiplimab, and one with the Aurora-A kinase inhibitor alisertib. Given the paucity of treatment options for relapsed or refractory MRT or RTK, these agents were selected based on the availability of clinical trials at the time of disease progression or recurrence and a biological rationale for using these agents for *SMARCB1*-deficient tumors (23–26). However, no objective responses were observed, and all five patients died of disease.

Despite these poor outcomes, there were long-term survivors in our series, indicating that certain patients with RTK and MRT can be successfully treated with intensive multimodal therapy. An important prognostic factor is stage or group at diagnosis. Our study demonstrated that outcomes were superior in patients with low stage disease compared to advanced stage, with 3-year EFS and OS of 75% for the five patients with stage/group I/II disease. Similarly, the COG AREN0321 study demonstrated 4-year EFS of 83% in six individuals with stage/group I/II disease (10). The results of AREN0321 were superior to the NWTS-5 study that used a less-intensive treatment regimen, suggesting that intensive chemotherapy may provide benefit to patients with low-stage disease. In the EPSSG-2005 trial, 4-year EFS was 68.6% for group I and 33.3% for group II (8).

Age at diagnosis has also been implicated as a prognostic factor. Sultan et al. evaluated 229 patients with both extracranial rhabdoid tumors and AT/RT, finding that patients diagnosed before age two years or after 18 years had significantly worse survival compared to those age 2–18 years (13). Similarly, in a retrospective study of 53 patients with MRT or RTK, Cheng et al. reported significantly better overall survival in patients diagnosed at age greater than one year compared to those diagnosed at age less than or equal to one year (27). The EuRhab Registry demonstrated poor outcomes for infants less than six months old with rhabdoid tumor at various sites (12). For our cohort, 3-year EFS was significantly higher in patients diagnosed at or after age two years compared to those diagnosed before age two years, consistent with previous studies and suggesting that younger age at diagnosis is associated with worse prognosis. Larger, multi-institutional studies are needed to clarify the impact of tumor site, stage, and age on outcomes.

Somatic mutations in *SMARCB1* are well-established drivers of MRT and RTK (28, 29) and the absence of INI1 protein staining by immunohistochemistry is pathognomonic for rhabdoid tumors. In our cohort, all patients demonstrated INI1 loss; two had somatic SMARCB1 mutations, and one had a 22q deletion. However, of the five patients tested for germline mutations in SMARCB1, only one patient was heterozygous for a pathogenic mutation in SMARCB1. The presence of a germline SMARCB1 mutation has implications for cancer screening, emphasizing the importance of molecular diagnostics and genetic screening in patients with rhabdoid tumors (30). The presence of a germline SMARCB1 mutation has also been associated with adverse prognosis in patients with MRT (12). Additional risk factors for rhabdoid tumors are currently under investigation. Heck et al. (31) reported associations between rhabdoid tumors and low birthweight, prematurity, late-term delivery, and multiple births, though further studies are necessary to better clarify these relationships and underlying mechanisms.

A strength of the study was the patient-specific data and detailed treatment information available, including second and third-line therapies. The limitation of the study is that it comprises a single-institution experience, limiting generalizability due to small sample size and lack of statistical power to detect differences among upfront treatment groups, disease stages at diagnosis, or treatment regimens. Given the small sample size, definitive conclusions cannot be drawn and larger, multi-institutional studies are needed, particularly in the relapsed or refractory disease setting.

In conclusion, our findings illustrate the limitations of intensive multimodal therapy, including regimens with ifosfamide and/or higher-dose cyclophosphamide, for RTK and MRT. Nevertheless, patients with low-stage disease had relatively favorable outcomes with intensive therapies. Development of effective and less toxic treatments remains an urgent priority for this rare malignancy.

## Data Availability

The original contributions presented in the study are included in the article/supplementary material. Further inquiries can be directed to the corresponding author.
